# The Predicted Key Molecules, Functions, and Pathways That Bridge Mild Cognitive Impairment (MCI) and Alzheimer's Disease (AD)

**DOI:** 10.3389/fneur.2020.00233

**Published:** 2020-04-03

**Authors:** Ye Tao, Yu Han, Lujiao Yu, Qi Wang, Sean X. Leng, Haiyan Zhang

**Affiliations:** ^1^Department of Geriatrics, The First Affiliated Hospital of China Medical University, Shenyang, China; ^2^Department of Neurology, Jinqiu Hospital of Liaoning Province, Shenyang, China; ^3^Division of Geriatric Medicine and Gerontology, Department of Medicine, Johns Hopkins University School of Medicine, Baltimore, MD, United States

**Keywords:** MCI, AD, WGCNA, PPI, GO, KEGG, microRNA, transcription factor

## Abstract

To elucidate the key molecules, functions, and pathways that bridge mild cognitive impairment (MCI) and Alzheimer's disease (AD), we investigated open gene expression data sets. Differential gene expression profiles were analyzed and combined with potential MCI- and AD-related gene expression profiles in public databases. Then, weighted gene co-expression network analysis was performed to identify the gene co-expression modules. One module was significantly negatively associated with MCI samples, in which gene ontology function and Kyoto Encyclopedia of Genes and Genomes pathway enrichment analysis showed that these genes were related to cytosolic ribosome, ribosomal structure, oxidative phosphorylation, AD, and metabolic pathway. The other two modules correlated significantly with AD samples, in which functional and pathway enrichment analysis revealed strong relationships of these genes with cytoplasmic ribosome, protein binding, AD, cancer, and apoptosis. In addition, we regarded the core genes in the module network closely related to MCI and AD as bridge genes and submitted them to protein interaction network analysis to screen for major pathogenic genes according to the connectivity information. Among them, small nuclear ribonucleoprotein D2 polypeptide (*SNRPD2)*, ribosomal protein S3a (*RPS3A)*, S100 calcium binding protein A8 (*S100A8)*, small nuclear ribonucleoprotein polypeptide G (*SNRPG)*, U6 snRNA-associated Sm-like protein LSm3 (*LSM3)*, ribosomal protein S27a (*RPS27A)*, and ATP synthase F1 subunit gamma (*ATP5C1)* were not only major pathogenic genes of MCI, but also bridge genes. In addition, *SNRPD2, RPS3A, S100A8, SNRPG, LSM3*, thioredoxin (*TXN)*, proteasome 20S subunit alpha 4 (*PSMA4)*, annexin A1 (*ANXA1)*, DnaJ heat shock protein family member A1 (*DNAJA1)*, and prefoldin subunit 5 (*PFDN5)* were not only major pathogenic genes of AD, but also bridge genes. Next, we screened for differentially expressed microRNAs (miRNAs) to predict the miRNAs and transcription factors related the MCI and AD modules, respectively. The significance score of miRNAs in each module was calculated using a hypergeometric test to obtain the miRNApivot-Module interaction pair. Thirty-four bridge regulators were analyzed, among which hsa-miR-519d-3p was recognized as the bridge regulator between MCI and AD. Our study contributed to a better understanding of the pathogenic mechanisms of MCI and AD, and might lead to the development of a new strategy for clinical diagnosis and treatment.

## Introduction

Alzheimer's disease (AD), a complex neurodegenerative disease and the most common cause of dementia, is characterized by brain atrophy, loss of synapses and neurons, amyloid plaques, and neurofibrillar tangles (NFTs) (1, 2). Clinical symptoms of AD include memory loss, daily living disorders, neuropsychiatric symptoms, and other behavioral disorders, all of which have serious effects on a patient's quality of life (3). Mild cognitive impairment (MCI) is an intermediate stage between normal brain aging and dementia, which is characterized by the relative preservation of basic daily function. MCI usually occurs before AD, and the difference between MCI and AD depends on the severity of cognitive decline that leads to functional impairment (4). MCI mainly includes amnesia MCI (aMCI), single domain non-amnesia MCI, and multiple domain MCI. Patients with aMCI and multiple domain MCI are at greater risk of developing AD, and the transition from MCI to AD can be conservatively estimated at 5–10% per year (5–8). Therefore, it is important to explore effective diagnosis methods for the early or prodromal stage of AD. The identification of non-invasive biomarkers for the rapid screening patients at high risk of progressing from MCI to AD would make a valuable contribution to guiding clinical treatment.

Weighted gene co-expression network analysis (WGCNA) is a bioinformatic analysis method that can determine the bridge between sample characteristics and gene expression changes (9). Previous studies had reported that WGCNA could be utilized to analyze the relationships between genes and relationships between gene expressions and clinical characteristics of neurodegenerative diseases (10–12). Six genes were recognized to be involved in the pathological changes of AD (11). It had been reported that 44 pathway pairs and 52 risk genes might participate in the pathological changes of PD (12). Protein-protein interactions (PPIs) are very important in most biological functions and processes (13) and mainly focus on detecting the relationship between protein complexes and functional modules (14).

The transcriptome of pan-cortical brain regions has been analyzed to screen the changes of gene expression related to the severity of AD. At the same time, region-specific co-expression networks and gene modules were constructed, which were correlated with disease characteristics and showed that changes to oligodendrocytes mainly occurred in the early stages of AD progression (15). Combining the brain-specific protein interaction group with the gene network demonstrated that there were extensive changes in the expression levels of different complex gene clusters in AD, among which overall expression was downregulated for gene associated with synaptic transmission, metabolism, cell cycle, survival, and immune response (16). Three new candidate genes screened by differential gene expression, gene ontology (GO) enrichment analysis, pathway analysis, and PPI analysis, were identified as potential candidates for AD pathology (17). These studies laid the foundation for understanding the potential pathogenesis and potential new treatment targets of MCI and AD.

MicroRNAs (miRNAs) are small non-coding RNAs with a length of approximately 22 nucleotides. MicroRNAs play important roles in regulating the expression of mRNAs, representing effective post-transcriptional regulators of gene expression (18). MicroRNAs can act as biomarkers for a variety of diseases, either alone or in combination with other known biomarkers. In addition, cells can secrete miRNAs through exocrine or extracellular vesicles, and the secreted miRNAs can remain stable in body fluids (19, 20). Six miRNAs (miR-483-5p, miR-486-5p, miR-30b-5p, miR-200a-3p, miR-502-3p, miR-142-3p) in plasma samples were able to support the diagnosis of possible early AD in patients with MCI compared with the control group (21). The expression of six different microRNAs (miR-181a-5p, miR-361-3p, miR-23a-3p, miR-15b-3p, miR-130a-3p, miR-27b-3p) is associated with a *SNAP25* (encoding synaptosome associated protein 25) polymorphism that could affect the neuroplasticity of the brain in patients with AD and had an influence on AD progression (22).

Currently, most studies have investigated pathogenic genes or biomarkers for a single stage of MCI or AD. The use of gene microarray technology has made great progress in clarifying the potential molecular mechanisms of MCI and AD. However, research on genes that play a vital role in the progression from MCI to AD is limited. Therefore, studying the key bridge molecules between MCI and AD is important for early diagnosis and treatment. The goal of this study was to analyze a dataset of 711 samples by using bioinformatic methods to analyze the differentially expressed genes in the public database. According to the connectivity information provided by WGCNA and PPI analyses, we screened major pathogenic genes, bridge genes, and bridge pathways. Then, the interaction data of miRNA-mRNA and mRNA-TF were downloaded from a public database. The miRNA and TF regulatory factors were predicted and the bridge regulators between MCI and AD were identified. The identified major pathogenic genes, bridge genes, bridge pathways, and bridge regulators might lead to novel therapeutic approaches for patients who are at higher risk of progressing from MCI to AD.

## Materials and Methods

### Microarray Data Preparation and Processing

The human whole blood mRNA expression data of the GSE63063 dataset (23) were downloaded from Gene Expression Omnibus (GEO) database (24). This dataset belonged to two platforms (GPL6947 and GPL10558). There were 329 samples in the dataset belonging to the GPL6947 platform, including 80 MCI samples, 145 AD samples, and 104 normal samples. There were 388 samples belonging to the GPL10558 platform, consisting of 109 MCI samples, 139 AD samples, 134 normal samples, 3 borderline MCI samples, 1 control (CTL) to AD sample, 1 MCI to CTL sample, and 1 other sample. We also downloaded 261 human MCI-related genes from The National Center for Biotechnology Information (NCBI)-Gene database (25) and the top 200 human MCI-related genes were downloaded from the Online Mendelian Inheritance in Man (OMIM) database (26). We also downloaded 544 human AD-related genes from the NCBI-Gene database and the top 200 human AD-related genes from the OMIM database. The miRNA expression data of the GSE120584 dataset (27) were downloaded from the GEO database, which included 1,021 AD samples, 32 MCI samples, and 288 normal samples. The protein interaction data were downloaded from The Human Protein Reference Database (HPRD) (28). MiRNA-mRNA interaction data were downloaded from the Starbase v2.0 database (29) and the mRNA-TF interaction data were downloaded from the TRRUST (transcriptional regulatory relationships unraveled by sentence-based text-mining) database (30).

### Analysis of Differentially Expressed Genes

The gene expression profile data of the two platforms (GPL6947 and GPL10558) in the GSE63063 dataset were integrated. Only MCI samples, AD samples, and normal samples were retained in the two platforms by removing borderline MCI samples, CTL to AD samples, MCI to CTL samples, and other samples from the GPL10558 platform. According to the platform annotation data, the probes of the two platforms are associated with the gene symbols. If one gene corresponded to multiple probes, the mean expression value of the probes was taken as the expression value of the gene. Surrogate Variable analysis (SVA 3.28.0) in the R package (31) was used to remove the batch effect between the two platforms and the gene expression profile was obtained after the batch effect was removed. The differentially expressed genes between MCI samples and normal samples (DEG1), the differentially expressed genes between AD samples and normal samples (DEG2), and the differentially expressed genes between MCI samples and AD samples (DEG3) were screened and obtained using the Limma 3.36.5 R package (32) for differential expression analysis (fold-change > 1.2 or fold-change <5/6, *p* < 0.05). The hierarchical clustering analysis (33) was done and DEGs were displayed in heat map and volcano plot.

### Analysis of WGCNA Co-expression

WGCNA was used to cluster genes into models or networks according to the weighted correlation coefficient between the genes and analyze the correlation between the module and the characteristics of the samples. The DEG1 data between MCI samples and normal samples and DEG3 data between MCI samples and AD samples and MCI-related genes in public databases were combined to obtain the potential genes associated with MCI and then used to construct the potential gene expression profile of MCI. Co-expression analysis was carried out using WGCNA in the R-package. The DEG2 data between AD samples and normal samples and DEG3 data between MCI samples and AD samples and AD-related genes in public databases were combined to obtain the potential genes associated with AD and then used to construct the potential gene expression profile of AD. Co-expression analysis was again carried out by WGCNA. The WGCNA algorithm was used to excavate the co-expressed gene modules, and the relationships between these modules and the phenotype of the sample were analyzed. Statistical significance is revealed by regression of characteristic genes of features and modules, also known as trait analysis (34), which reveals the modules that are significantly associated with disease (*P* < 0.05). The correlation between CTL and disease is examined with Pearson's correlation coefficient. Cytoscape was used to identify the MCI- and AD-related modules for network display (35).

### Functional Enrichment Analysis of Module Genes and Identification of Bridge Function, Pathway, Genes, and Pathogenic Major Genes

EnrichR 1.0 in the R package (36) was used to analyze the function and pathway enrichment of the genes in the MCI- and AD-related modules. The intersections of the functions and pathways in the two groups of modules was regarded as the bridge functions and pathways. The genes in the MCI- and AD-related modules was intersected and the core genes in these two types of module network were identified and termed as the bridge genes. The protein interaction data was downloaded from the HPRD database. The genes in the MCI-related modules and the AD-related modules were subjected to PPI analysis to determine their connectivity. We identified the genes with high connectivity as major pathogenic genes and the connectivity of bridge genes was observed.

### Screening for Differentially Expressed miRNAs

The differentially expressed miRNAs between the MCI samples and normal samples, and between AD samples and normal samples, were screened using the R software package Limma for differential expression analysis (fold-change > 1.2 or foldchange <5/6, *p* < 0.05).

### Prediction and Analysis of Regulatory Factors of microRNAs and TFs to Identify Bridge Regulators

By taking the human miRNA-mRNA interactions included in the Starbase as the interaction background, we looked for miRNAs that could regulate the functional modules of MCI and AD. Based on the 9,396 pairs of human TF-mRNA regulatory relationships recorded in the TRRUST v2 database, the TFs that could regulate the functional modules of MCI and AD were identified. Cytoscape was applied to visualize the interaction between the miRNAs and TFs and the modules. The intersection of the regulatory factors (miRNA, TF) of the MCI- and AD-related modules were termed bridge regulators.

## Results

### Data Preprocessing

First, the gene expression profile data of the two platforms (GPL6947 and GPL10558) of the GSE63063 dataset were integrated to retain only MCI samples, AD samples, and normal samples by removing borderline MCI samples, CTL to AD samples, MCI to CTL samples, and other samples. Finally, a total of 711 samples were obtained from the two platforms, including 189 MCI samples, 284 AD samples, and 238 normal samples. The samples on the GPL6947 platform were annotated to 29,957 genes and the samples on the GPL10558 platform were annotated to 24,899 genes. The genes common to the two platforms (19,460 genes) were extracted to construct the expression profile of the shared genes between the two platforms.

**Figure 1:**
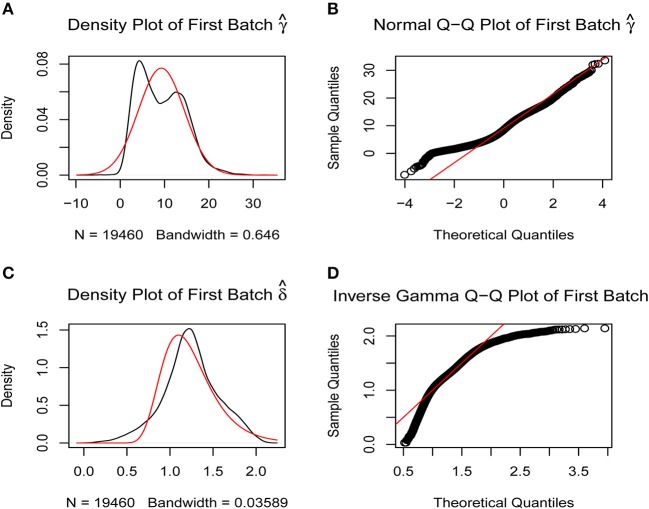
Removal of batch effects. The closer the scatter is to the straight line, the smaller the batch effect is. **(A,B)** Batch effects between the two platforms were corrected using SVA R package by normal Q-Q plot. **(C,D)** Batch effects between the two platforms were corrected using SVA R package by inverse gamma Q-Q plot.

A batch effect between the data from two platforms was identified and removed using the sva R package (Figures 1A–D) to obtain the final gene expression profile for further analysis.

### Screening of Differentially Expressed Genes

**Figure 2:**
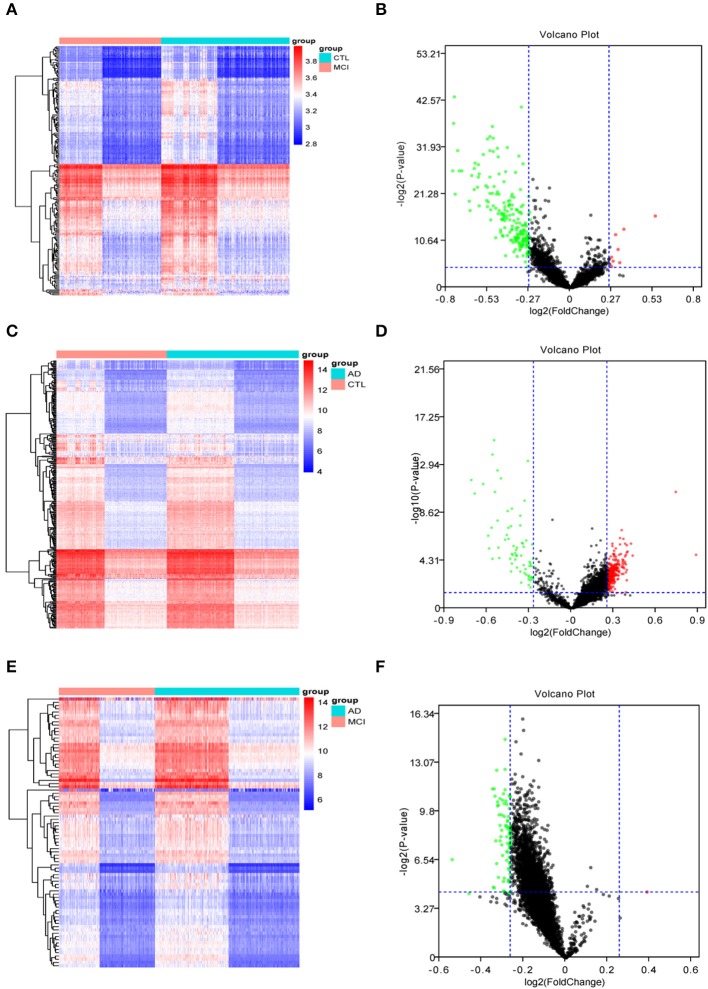
The heat maps and volcano plots of differentially expressed genes (DEGs) were obtained by cluster analysis. The horizontal axis represents the specimen in heat maps: CTL represents the normal sample, MCI represents the mild cognitive impairment sample, and AD represents the Alzheimer's disease sample. Red indicates genes that are up-regulated, while blue indicates genes that are down-regulated in heat maps. Red indicates genes that are up-regulated, while green indicates genes that are down-regulated in volcano plots. **(A,B)** The heat map and volcano plot of differentially expressed genes between MCI samples and normal samples. **(C,D)** The heat map and volcano plot of differentially expressed genes between AD samples and normal samples. **(E,F)** The heat map and volcano plot of differentially expressed genes between MCI and AD samples.

The Limma R package identified 205 DEGs between 189 MCI samples and 238 normal samples, of which eight were upregulated and 197 were down-regulated in MCI (DEG1, Table S1). The heat map of the DEG1 dataset is shown in Figure 2A and a volcano plot is shown in Figure 2B.

Similarly, Limma identified 315 DEGs between 284 AD samples and 238 normal samples, of which 247 genes were upregulated and 68 genes were downregulated in AD (DEG2, Table S2). The heat map of the DEG2 data is shown in Figure 2C and the volcano plot appears in Figure 2D.

Finally, 83 DEGs were identified among 189 MCI samples and 284 AD samples, of which 1 was upregulated and 82 were downregulated in MCI (DEG3, Table S3). Thus, the difference between the MCI samples and AD samples was significantly smaller than that between the disease samples and the normal samples. The heat map of the DEG3 data is shown in in Figure 2E and the volcano plot is shown in Figure 2F.

### WGCNA Co-expression Analysis

**Figure 3:**
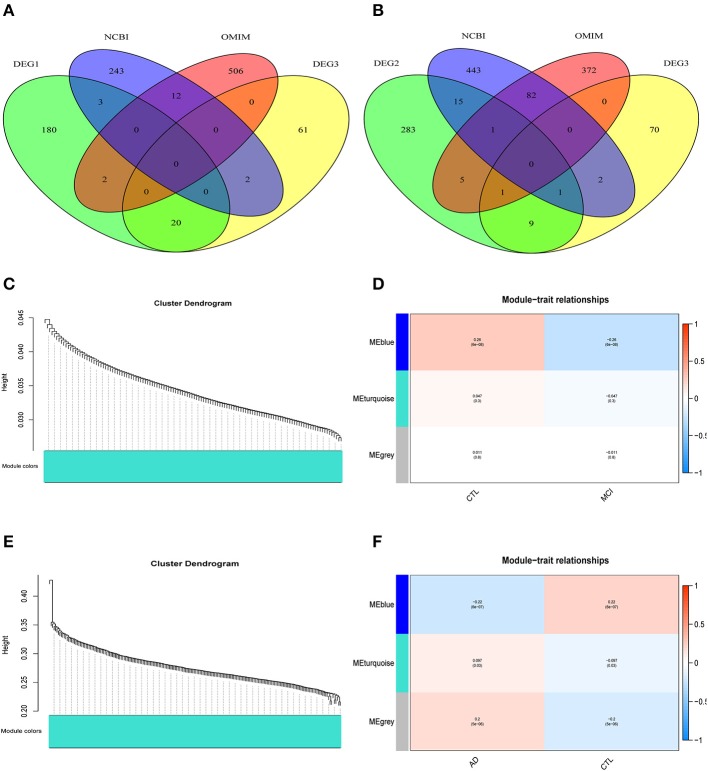
WGCNA co-expression network construction. **(A)** A total of 1029 potential MCI genes were obtained and displayed in venn figure. **(B)** A total of 1284 potential AD genes were obtained and displayed in venn figure. **(C)** The systematic tree diagram of module clustering for MCI genes. **(D)** WGCNA modules of MCI: each line corresponds to a gene co-expression network (marked with color). The numbers in the table represent the Pearson correlation coefficient *r* and the associated *p*-value in parentheses. **(E)** The systematic tree diagram of module clustering for AD genes. **(F)** WGCNA modules of AD: each line corresponds to a gene co-expression network (marked with color). The numbers in the table represent the Pearson correlation coefficient *r* and the associated *p*-value in parentheses.

The differentially expressed genes in DEG1 screened between MCI samples and normal samples and those in DEG3 screened between MCI samples and AD samples were combined with the MCI related genes in the public database (NCBI, OMIM) (a Venn diagram is shown in Figure 3A). A total of 1,029 potential MCI genes were obtained, and the potential gene expression profiles of MCI were constructed. Co-expression analysis was carried out by using R-package WGCNA.

The differentially expressed genes in DEG2 screened between AD samples and normal samples and those in DEG3 screened between MCI samples and AD samples were combined with the AD related genes in the public database (NCBI, OMIM) (a Venn diagram is shown in Figure 3B). A total of 1,284 potential AD genes were obtained, and the potential gene expression profiles of AD were constructed. Co-expression analysis was carried out by using R-package WGCNA.

Through the analysis of WGCNA co-expression, three modules were excavated from the potential gene expression profile of MCI. Figure 3C shows the systematic tree diagram of module clustering. The relationship between the three MCI modules and traits (normal, disease) is displayed in Figure 3D. The gene expression level in the blue module was significantly negatively correlated with the disease characteristics of MCI (*r*^2^ = −0.26, *p* = 6e^−08^), implying that the blue module could be used as the core module of MCI. Three modules were also excavated from the potential gene expression profile of AD. The systematic tree diagram of AD module clustering is shown in Figure 3E. The relationship between AD-related modules and traits is showed in Figure 3F, which suggested that the genes in the blue module were negatively related to the disease characteristics of AD (*r*^2^ = −0.22, *p* = 6e^−7^), whereas the genes in the turquoise module were positively associated with the disease characteristics of AD (*r*^2^ = 0.097, *p* = 0.03). Therefore, the blue and turquoise modules could be utilized as the core module of AD.

### Module Network Construction

**Figure 4:**
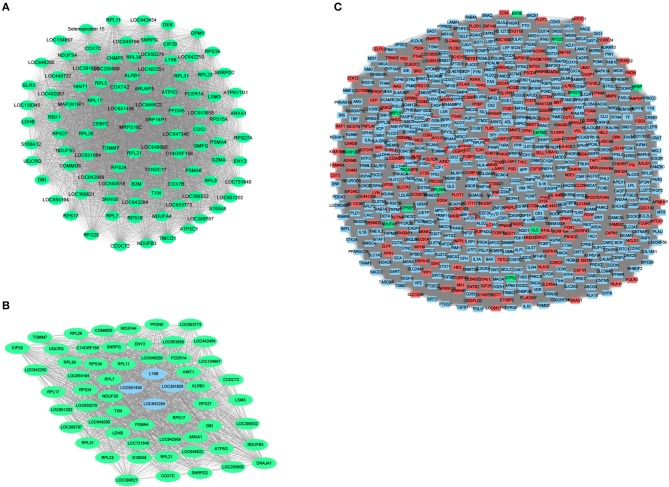
Module network construction. **(A)** The network diagram of the MCI blue module (green for down-regulated genes, red for up-regulated genes). **(B)** The network diagram of the AD blue module (green for down-regulated genes, red for up-regulated genes). **(C)** The network diagram of the AD turquoise module (green for down-regulated genes, red for up-regulated genes).

To further identify related modules for network display, we used Cytoscape to visualize the gene network of the relevant modules of MCI and AD, respectively. The network diagram of the MCI blue module is shown in Figure 4A, the network diagram of the AD blue module is shown in Figure 4B, and the network diagram of the AD turquoise module is shown in Figure 4C (green for downregulated genes and red for upregulated genes). We demonstrated that all the genes in the MCI blue module network diagram were differentially expressed, indicating that the DEGs were indeed related to the characteristics of MCI. We also concluded that most genes in AD blue module network diagram was differentially expressed. Moreover, there were also differentially expressed genes in the AD turquoise module, implying that the differentially expressed genes were indeed associated with the characteristics of AD.

### Functional Enrichment Analysis of Modular Genes and the Identification of Bridge Functions and Pathways

**Figure 5:**
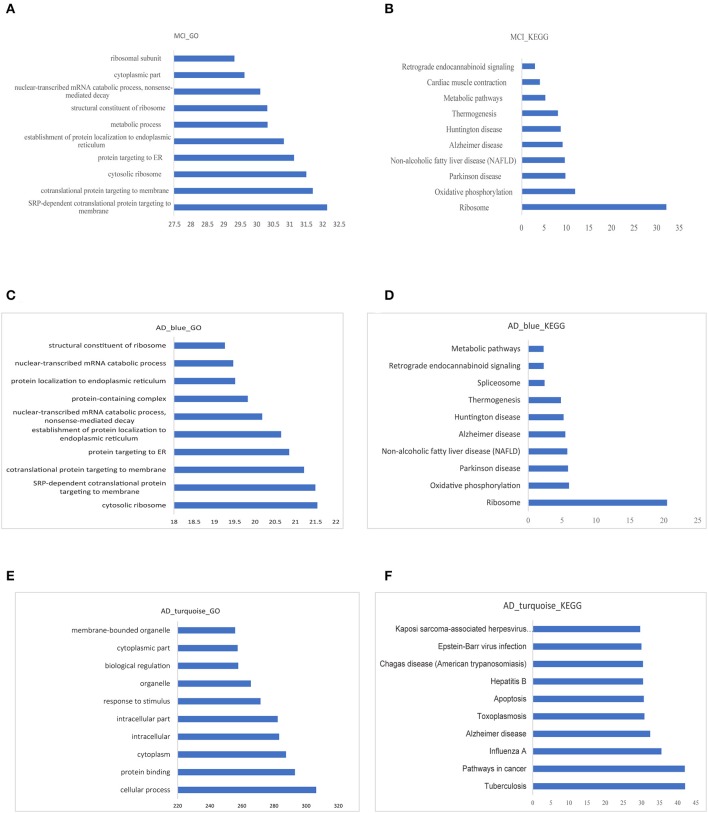
Functional enrichment analysis of modular gene. **(A,B)** The 10 most significant GO functions and KEGG pathways of MCI blue module. **(C,D)** The 10 most significant GO functions and KEGG pathways of AD blue module. **(E,F)** The 10 most significant GO functions and KEGG pathways of AD turquoise module.

The GO functional enrichment analysis and Kyoto Encyclopedia of Genes and Genomes (KEGG) pathway enrichment analysis of the related module genes of MCI and AD were carried out by using g:profile. Top 10 GO functional enrichment analysis diagram and KEGG pathway enrichment diagram of the MCI-related blue module and the AD related blue and turquoise modules are displayed in Figures 5A–F. The results of enrichment analysis suggested that most of the MCI blue module genes were enriched in several GO terms, such as (signal recognition particle) SRP-dependent cotranslational protein targeting to membrane and cytosolic ribosome. Besides, the result of KEGG analysis revealed ribosome and oxidative phosphorylation as significant enriched pathways in MCI. Cytosolic ribosome, SRP-dependent cotranslational protein targeting to membrane and protein targeting to ER (endoplasmic reticulum) were mainly enriched GO terms of AD blue module genes. In additon, the result of KEGG analysis of AD blue module genes showed ribosome was the most significant enriched pathway. Whereas, AD turquoise module genes mostly demonstrated a highly significant enrichment of AD, cancer-related pathways, and apoptosis in KEGG analysis. Previous studies have reported that several ribosomal protein genes were downregulated in the hippocampus of patients with AD at first stage (I–II) preceding neuron loss, which are regulated by nucleolar stress (37). In fact, MCI and AD blue module genes were enriched in many similar pathways and functions, especially in ribosome-related biological processes, which implied that ribosome dysfunction might participate in the pathogenesis and progression of both MCI and AD.

The intersection of all the GO functions in MCI and AD were used as bridge functions and a total of 326 bridge functions were obtained (Table S4). Moreover, we analyzed the intersection of all the KEGG pathways with significant enrichment in MCI and AD as bridge pathways, which identified 10 bridge pathways (Table S5).

### Identification of Bridge Genes

The genes in the MCI related blue module and the AD related modules (blue, turquoise) were intersected, which were regarded as the overlapping genes (*n* = 61). Next, the core genes in the two disease module networks were identified as crosstalk genes (Table S6). The degree of gene nodes with a connection threshold >0.3 in the MCI blue module and AD blue module network were calculated and the top 40 genes were utilized as candidate crosstalk genes. The degree of the gene node with a connection threshold >0.6 in the AD related turquoise module network was calculated and the genes with the highest screening degree were used as the crosstalk genes of the candidate AD turquoise module. Finally, 123 crosstalk genes were obtained from MCI module (blue) and AD module (blue, turquoise). By selecting overlapping genes and crosstalk genes as bridge genes, we identified 120 bridge genes (Table S7).

### Identification of Major Pathogenic Genes

**Figure 6:**
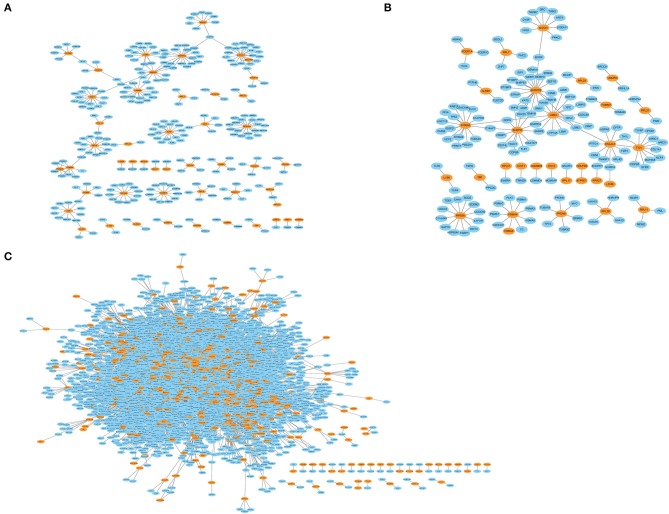
Protein interaction network (PPI). Orange represents module genes, blue represents PPI genes. **(A)** The genes in the MCI-related blue module were put into the network map of the PPI. **(B)** The genes in the AD-related blue module were put into the network map of the PPI. **(C)** The genes in the AD-related turquoise module were put into the network map of the PPI.

The genes in the MCI related module (blue) and AD related modules (blue, turquoise) were subjected to a PPI connectivity analysis. Genes with high connectivity were identified as major pathogenic genes and the connectivity of bridge genes was observed. As shown in Figures 6A,B, the genes in the MCI-related blue module and AD-related blue module were placed into the network map of the PPIs. The genes in the AD-related turquoise module were placed into the network map of the PPIs, as shown in Figure 6C. Then, the top 10 genes of the MCI_PPI network connectivity were selected as the major genes of MCI, among which, *SNRPD2, RPS3A, S100A8, SNRPG, LSM3, RPS27A*, and *ATP5C1*were not only major pathogenic genes of MCI, but also bridge genes (Table 1). Furthermore, we selected the top 10 genes of the AD_PPI network connectivity as the major genes of AD, among which, *SNRPD2, RPS3A, S100A8, SNRPG, LSM3, TXN, PSMA4, ANXA1, DNAJA, and PFDN5* were not only major pathogenic genes of AD, but also bridge genes (Table 1). In addition, *SNRPG, RPS3A, LSM3, S100A8*, and *SNRPD2* were common major pathogenic genes of both MCI and AD.

**Table 1:** Bridge genes in major pathogenic genes of MCI and AD (The common bridge genes are shown in bold).

**Disease**	**Gene**	**Upregulated or downregulated in diseases**	***P*-value**
**MCI**	**SNRPD2**	Down	3.68E-13
	**RPS3A**	Down	6.79E-10
	**S100A8**	Down	6.65E-09
	**SNRPG**	Down	1.82E-07
	**LSM3**	Down	3.38E-07
	RPS27A	Down	5.44E-07
	ATP5C1	Down	4.48E-06
**AD**	**SNRPD2**	Down	4.44E-14
	**RPS3A**	Down	2.37E-07
	PFDN5	Down	3.18E-06
	**LSM3**	Down	9.59E-06
	**S100A8**	Down	1.31E-05
	TXN	Down	1.76E-05
	PSMA4	Down	6.52E-05
	**SNRPG**	Down	8.85E-05
	DNAJA1	Down	0.000116
	ANXA1	Down	0.007498

### Screening of Differentially Expressed miRNA

The Limma R package was used to screen differentially expressed miRNAs (DEmiRNA_AD) between 1,021 AD samples and 288 normal samples in the miRNA expression data of GSE120584 dataset. We also identified differentially expressed miRNAs (DEmiRNA_MCI) between 32 MCI samples and 288 normal samples. As a result, 178 differentially expressed miRNAs were obtained between AD samples and normal samples (Table S8) and 218 differentially expressed miRNAs were obtained between MCI samples and normal samples (Table S9).

### Factors With Predicted Regulatory Functions on the Modules (miRNA, TF)

The interaction between human miRNAs and mRNA was obtained from the Starbase 2.0 database. The miRNAs that are important in regulating the MCI functional modules (blue) and AD functional modules (blue, turquoise) were selected to calculate the significance scores of miRNAs and modules using the hypergeometric test (*p* < 0.05), under the conditions that at least two genes interacted with each other. The interaction between human TFs and mRNA were obtained from TRRUST database. Next, we selected the important TFs regulating MCI functional modules (blue) and AD functional modules (blue, turquoise) to calculate the significance scores of the TFs and modules using the hypergeometric test (*p* < 0.05), under the conditions that at least two genes interacted with each other.

**Figure 7:**
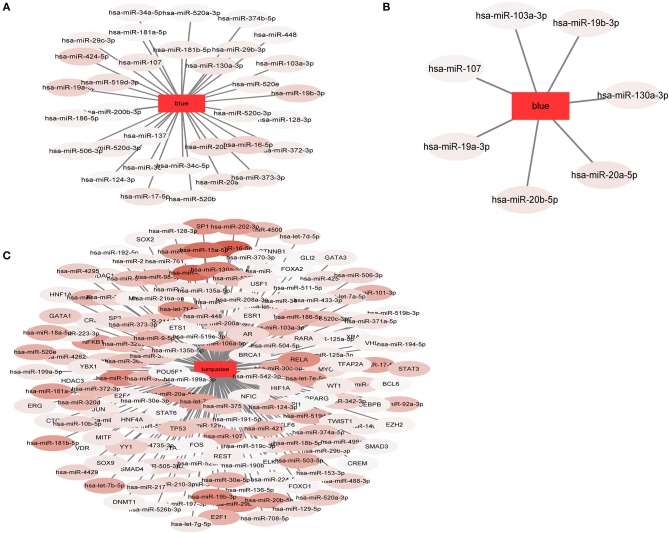
The network diagram of the relationship between the module and the regulatory factors. The rectangle represents the module and the oval represents the regulator. The darker the color of the regulatory factor, the more significant the *P*-value of the interaction. **(A)** 34 miRNApivot-Module interaction pairs were obtained in MCI blue module. **(B)** 7 miRNApivot-module interaction pairs were obtained in AD blue module. **(C)** 118 miRNApivot-module interaction pairs and 61 TFpivot-module interaction pairs were obtained in AD turquoise module.

In our study, 34 miRNApivot-Module interaction pairs were obtained for the MCI blue function module; however, no significant TFpivot-Module interaction pairs were identified (Table S10). The network diagram of the relationship between the MCI module and the regulatory factors is shown in Figure 7A. For the AD blue function module, seven miRNApivot-module interaction pairs were obtained; however, no significant TFpivot-module interaction pairs were identified. We constructed the network diagram of the relationship between the AD blue module and the regulatory factors in Figure 7B. Nevertheless, we performed 118 miRNApivot-module interaction pairs and 61 TFpivot-module interaction pairs for the AD turquoise function module (Table S11). The network diagram of the relationship between the AD turquoise module and the regulatory factors is displayed in Figure 7C.

### Identification of Bridge Regulators

For more direct analysis, the intersection of the regulatory factor (pivot) of the MCI module and the AD module was used as the bridge regulator, and a total of 34 bridge regulators were obtained (Table S12). Among the 34 bridge regulators screened, hsa-miR-519d-3p was not only a bridge regulator, but also a differentially expressed miRNA between the MCI and normal samples.

## Discussion

**Figure 8:**
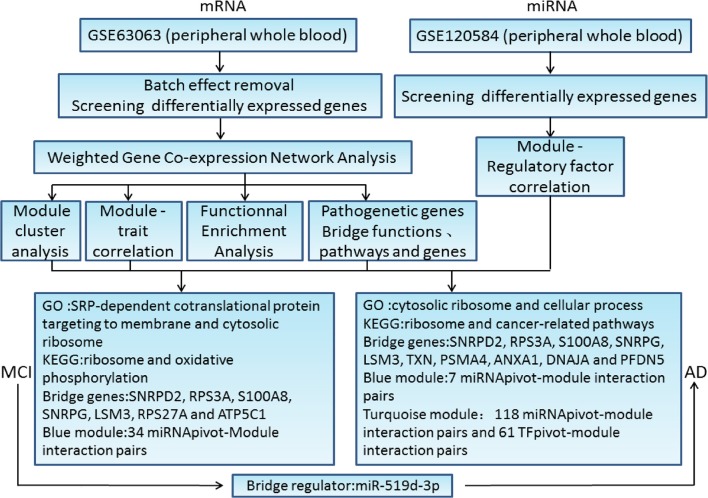
The major workflow and conclusions.

Our present study was aimed to find the common characteristics of MCI and AD by integrating research data from these two diseases to explore the potential gene expression profile. The major workflow and conclusions were showen in Figure 8. A total of 711 samples, including those from patients with MCI and AD, and normal controls, were analyzed systematically. By screening the association of DEGs with disease-related genes in public databases, 1,029 potential MCI genes and 1,284 potential AD genes were identified and subjected to WGCNA co-expression analysis. The modules significantly related to the disease characteristics were constructed and analyzed. Functional enrichment analysis revealed 326 bridge functions, 10 bridge pathways, and 120 bridge genes. MCI module genes were mostly enriched in cytosolic ribosome, SRP-dependent cotranslational protein targeting to membrane, oxidative phosphorylation, ribosomal structure, Parkinson's syndrome, AD, and metabolism. Meanwhile, AD module genes showed a significant enrichment of cytosolic ribosome, SRP-dependent cotranslational protein targeting to membrane, protein targeting to ER, protein binding, AD, apoptosis, and cancer-related pathways and functions. Previous studies had reported that differentially expressed genes between AD patients and normal brain tissues were mainly enriched in oxidative phosphorylation, Parkinson's disease, protein transport, Alzheimer's disease and SRP-dependent cotranslational protein targeting to membrane, etc. (11, 38, 39), which are generally consistent with our findings. The enrichment of AD genes in cancer-related pathways reveal that some degrees of overlap in the potential pathogenesis of AD and cancers. Genes associated with neurodegenerative diseases, such as PD, are often abnormally expressed in cancers and also involved in cell cycle maintenance (12). Therefore, genes involved in cancers may be potential therapeutic targets for AD. Indeed, MCI and AD are enriched in many similar functions and pathways, which might be important for the pathogenesis and progress from MCI to AD.

According to our results, *ATP5C1, RPS27A, SNRPG, LSM3, RPS3A, S100A8*, and *SNRPD2* were not only major pathogenic genes of MCI, but also bridge genes. Furthermore, *SNRPG, LSM3, RPS3A, S100A8, SNRPD2, ANXA1, DNAJA1, PFDN5*, and *PSMA4* were not only major pathogenic genes of AD, but also bridge genes. In addition, a total of 34 bridge regulators were obtained, among which, hsa-miR-519d-3p was a differentially expressed miRNA between MCI and normal samples and was identified as a bridge regulator between MCI and AD. Our findings support the idea that changes in peripheral blood are associated with progression from MCI to AD.

In our study, *ATP5C1* and *RPS27A* were regarded as major pathogenic genes of MCI, which is consistent with the conclusions that mitochondrial dysfunction and oxidative stress are closely associated with progression of cognitive impairment (40). The increased accumulation of Aβ caused by mitochondrial dysfunction might be strongly related to the occurrence and progress of AD. We found that mitochondrial F1 complex *ATP5C1* was downregulated in MCI, which could lead to mitochondrial dysfunction (41), suggesting that *ATP5C1* may be a potential diagnostic and therapeutic target. Oxidative stress can cause transiton of cell cycle from the stationary G0 phase to irregular DNA replication and mitosis by causing DNA damage in neurons (42), which leads to tau overphosphorylation and aggregation into NFTs probably (43). It is worth emphasizing that cell cycle-related proteins exhibit abnormal expression in hippocampal neurons of patients with MCI (44, 45). *RPS27A* is synthesized as a C-terminal extension of ubiquitin, while ubiquitin is involved in DNA repair, cell-cycle regulation, and protein degradation via the proteasome. The expression of *RPS27A* is specifically induced in the middle and late G1 phase of the cell cycle, mainly to ensure that the biological process of the cell cycle proceeds smoothly (45). We recognized *RPS27A* showed decreased expression level comparing with normal samples, indicating that reduced *RPS27A* expression might be involved in the development of MCI through conducting to arrest of cell cycle.

Recent studies demonstrated that hippocampal atrophy in patients with AD is mainly secondary to neurofibrillar tangles formation, and neuronal atrophy or loss. However, the main reason for hippocampus atrophy may be the decrease of protein synthesis. In fact, in the early stage of AD, nucleolar stress changes the level of ribosomal gene expression, which leads to changes in the ribosomal composition and damage to protein synthesis in the cortical region of patients with MCI or those with dementia caused by AD. The main manifestations are decreased expression levels of rRNA and ribosomal proteins, decreased ability of isolated polyribosomes to bind S-methionine to proteins, and changes in the expression of ribosomal protein genes before cell death (37, 46).

We identified *SNRPG, LSM3, RPS3A, S100A8*, and *SNRPD2* as major pathogenic genes of both MCI and AD. During the occurrence and progression of MCI and AD, several genes are enriched in ribosomal structure and function. Ribosomes are complexes composed of nucleic acids and proteins that are responsible for mediating the synthesis of all proteins, and comprise specialized nucleic acids, rRNA, and tRNA molecules, which are necessary for ribosomes to convert mRNA into proteins (47, 48). During protein synthesis, rRNA and tRNA levels decreased and rRNA oxidation was increased in the cognition related brain regions of patients with MCI (46). Nevertheless, the expression levels of transcription factors leading to a reduction in protein synthesis showed no significant changes, implying that decreased protein synthesis might be strongly associated with ribosomal dysfunction in the early stage of MCI. *SNRPG* belongs to the small nuclear ribonucleoprotein peptide family, which can directly bind to the Sm site of small nuclear RNA (snRNA) to form a variety of snRNP cores needed for RNA maturation, including pre-mRNA splicing, mRNA degradation, and telomere formation (49). We identified decreased expression level *of SNRPG*, which might participate in the progression from MCI to AD. In our study, *LSM3* was another major pathogenic gene, showing reduced expression in MCI and AD. *LSM3* is the most critical activating factor for mRNA removal in eukaryotic cells and participates in all steps of RNA metabolism, including RNA silencing and degradation (50, 51). Therefore, abnormal expression of *LSM3* might contribute to cognitive impairment. *RPS3A* is a ribosome chaperone protein in mammals, which was downregulated in both MCI and AD. When nerve cells become aged, the protective effect of *RPS3A* might be reduced, which results in α-synaptic nucleoprotein becoming increasingly toxic and leads to the eventual progression of neurodegenerative diseases (52). Moreover, *RPS3A* can prevent apoptosis by inhibiting poly ADP-ribose polymerase (PARP) activity, together with B-cell lymphoma-2 (BCL2) (53). Recent studies have shown that one of the R*PS3A* homologous genes was significantly associated with delayed AD (LOAD) in case-control studies, suggesting that *RPS3A* or its homologs are associated with adjacent genes or other functional variants, and play a crucial role in the pathogenesis of AD (54).

The severity of cognitive impairment is also related to synaptic and neuronal inflammatory injury (2). Thus, how to effectively intervene in neuroinflammation has become a new therapeutic target for MCI and early AD. We defined *S100A8* as a major pathogenic gene of both MCI and AD, which has higher connectivity in MCI and AD PPI networks. An increased expression level of *S100A8* aggravated neuronal inflammation by promoting the formation of amyloid β (Aβ) plaques and showed co-localization with Aβ plaques, which was compatible with the activation of astrocytes in the brains of APP23 mice, a mouse model of AD (55). However, our results showed decreased expression of *S100A8* in venous blood of patients with MCI or AD, which may be due to the tissue specificity of *S100A8* expression level and needs more investigation. Our results also identified *SNRPD2* as a major pathogenic gene of MCI and AD, with decreased expression level. The interaction of *SNRPD2* with nuclear retention elements significantly inhibits the export of long non-coding RNAs (lncRNAs) and mRNAs from the nucleus to the cytoplasm and knockdown of *SNRPD2* leads to an increase in the cytoplasmic distribution of endogenous lncRNAs (56), which might correlate with the pathogenesis from MCI to AD; however, further study is needed.

There are several genes identified as (47, 49–54) major pathogenic gene of AD, including *ANXA1, DNAJA1, PFDN5*, and *PSMA4. ANXA1* was reported to be associated with the early stage of AD in patients and animal models. By inhibiting the secretion of inflammatory mediators stimulated by Aβ, *ANXA1* could stimulate microglial phagocytosis of Aβ and reduce the level of Aβ (57). Our study revealed that *ANXA1* had decreased expression in patients with MCI and AD, indicating that a lower level of *ANXA1* might contribute to the increased degree of neuroinflammation and cognitive impairment. Thus, abnormal expression levels of inflammation-related factors are likely to participate in the progression of MCI and AD. In addition to the inflammatory mechanism, the protein toxicity mechanism mediated by the Aβ-peptide oligomer is also considered a key factor in the pathogenesis and progression of MCI and AD. The essence of Aβ is the aggregation of misfolded peptides. In the present study, we demonstrated that the genes related to Aβ misfolding, aggregation, or degradation were abnormally expressed to some extent. *DNAJA1*, also known as heat shock protein 40 (HSP40 or HSC40), can preserve misfolded proteins or polypeptide chains, and transport peptides through the membrane. By regulating the assembly and decomposition of protein complexes, *DNAJA1* mainly prevents the aggregation of misfolded polypeptide chains (58). Our results showed that *DNAJA1* was decreased in AD and might lead to the increased level of Aβ. *PSMA4* encodes a proteasome core structural protein and had decreased expression level in patients with AD. *PSMA4* can selectively regulate the expression level of intracellular protein and promote the degradation of misfolded proteins, and is expected to become a new target for the therapy of MCI and early AD (59). The *PFDN* family is expressed in a variety of tissues in eukaryotes, and could bind and stabilize unfolded target peptides and deliver newly synthesized peptides to II group chaperones (molecular chaperones) to complete the folding process and prevent misfolding (60). *PFDN5* is highly expressed in nerve cells and can protect cells from cell death induced by aggregated proteins and decreases the toxicity of misfolded proteins (61, 62). In addition, *PFDN5* indirectly reduces ribosomal biosynthesis by inhibiting the transactivation of c-myc, the main regulator of ribosomal biosynthesis (63, 64). Our results identified *PFDN5* had decreased expression level in AD, which might contribute to the increased toxicity of Aβ and ribosomal dysfunction. *PFDN5* might become a new biomarker for the diagnosis and treatment of AD.

Oxidative stress plays an important role in the pathological process of most neurodegenerative diseases, including AD. Oxidative stress induced by Aβ has adverse effects on nerve transmission, synaptic function, and cognitive function (65). Previous studies have reported that the levels of oxidative stress in patients with MCI were significantly higher than those in patients with AD and healthy samples, whereas the antioxidant capacity of patients with MCI and AD were similar (66, 67). High levels of oxidative stress might be a key factor leading to the development from MCI to AD. *TXN* (TRX1) plays a protective role in maintaining the intracellular environment and antioxidant capacity, which is involved in many redox reactions (68). Compared with control samples, the level of *TRX1* decreased significantly in the middle frontal cortex and hippocampal CA1 area of patients with AD (69, 70), which was clearly consistent with our research. We identified *TRX1* as a major pathogenic gene of AD, which was downregulated and implying that low expression levels of *TRX1* might lead to the development of AD (48).

In the present study, miRNA hsa-miR-519d-3p was identificated to be differentially expressed (upregulated) between MCI and normal samples, implying it as a bridge regulator between MCI and AD. Overexpression of miR-519d-3p inhibited the growth of pancreatic cancer cells by inhibiting expression of ribosomal protein (71). In addition, miR-519d-3p plays an important role in the occurrence and development of many kinds of tumors, including inhibiting the proliferation of laryngeal squamous cell carcinoma (72), inhibiting the proliferation and migration of colorectal cancer (73), inhibiting the invasion of gastric cancer (74), and promoting the apoptosis of cervical cancer cells (75). Furthermore, previous studies revealed that downregulation of miR-519d-3p could reduce the cardiomyocyte apoptosis induced by hypoxia (76). Taken together, these findings implied that a low expression level of miR-519d-3p might play a protective role in nerve cells.

There have been no previous reports of miR-519d-3p being involved in the pathogenesis of MCI and AD. Considering the increased expression of miR-519d-3p in MCI, we hypothesized that miR-519d-3p might inhibit the expression of a ribosomal protein in neurons, which in turn affected the level of protein synthesis. Furthermore, reduction of ribosomal function and protein synthesis has a negative impact on cognitive function. Thus, abnormal expression levels of miR-519d-3p might play a key biological role in the progression from MCI to AD. MicroRNA-519d-3p is expected to become a new biomarker to screen for those patients with MCI that are more likely to progress to AD and to guide clinical treatment.

## Conclusion

In summary, this present study revealed many bridge genes and pathways related to MCI and AD, and the related gene and pathway network could be used to guide further research to explain the molecular mechanism of MCI and AD. Some bridge genes, major pathogenic genes, and bridge regulators might also be potential therapeutic targets, although more comprehensive investigations are needed to verify the role of these genes in the development of MCI and AD.

## Data Availability Statement

All datasets generated for this study are included in the article/Supplementary Material.

## Author Contributions

HZ and SL: conception and design and administrative support. YT and YH: provision of study materials. YT and LY: collection and assembly of data. QW, YT, and YH: data analysis and interpretation. All authors: manuscript writing and final approval of manuscript.

### Conflict of Interest

The authors declare that the research was conducted in the absence of any commercial or financial relationships that could be construed as a potential conflict of interest.
